# Central nervous system exposure of next generation quinoline methanols is reduced relative to mefloquine after intravenous dosing in mice

**DOI:** 10.1186/1475-2875-10-150

**Published:** 2011-06-06

**Authors:** Geoffrey S Dow, Erin Milner, Ian Bathurst, Jayendra Bhonsle, Diana Caridha, Sean Gardner, Lucia Gerena, Michael Kozar, Charlotte Lanteri, Anne Mannila, William McCalmont, Jay Moon, Kevin D Read, Suzanne Norval, Norma Roncal, David M Shackleford, Jason Sousa, Jessica Steuten, Karen L White, Qiang Zeng, Susan A Charman

**Affiliations:** 1 Division of Experimental Therapeutics, Walter Reed Army Institute of Research, Silver Spring, MD, USA; 2 Medicines for Malaria Venture, International Center Cointrin, Route de Pre-Bois 20, PO Box 1826, CH-1215 Geneva 15, Switzerland; 3 Centre for Drug Candidate Optimisation, Monash Institute of Pharmaceutical Sciences, Monash University, 381 Royal Parade, Parkville, VIC 3052, Australia; 4 Division of Biological Chemistry and Drug Discovery, College of Life Sciences, University of Dundee, Sir James Black Centre, Dundee, DD1 5EH, Scotland, UK

## Abstract

**Background:**

The clinical use of mefloquine (MQ) has declined due to dose-related neurological events. Next generation quinoline methanols (NGQMs) that do not accumulate in the central nervous system (CNS) to the same extent may have utility. In this study, CNS levels of NGQMs relative to MQ were measured and an early lead chemotype was identified for further optimization.

**Experimental design:**

The plasma and brain levels of MQ and twenty five, 4-position modified NGQMs were determined using LCMS/MS at 5 min, 1, 6 and 24 h after IV administration (5 mg/kg) to male FVB mice. Fraction unbound in brain tissue homogenate was assessed *in vitro *using equilibrium dialysis and this was then used to calculate brain-unbound concentration from the measured brain total concentration. A five-fold reduction CNS levels relative to mefloquine was considered acceptable. Additional pharmacological properties such as permeability and potency were determined.

**Results:**

The maximum brain (whole/free) concentrations of MQ were 1807/4.9 ng/g. Maximum whole brain concentrations of NGQMs were 23 - 21546 ng/g. Maximum free brain concentrations were 0.5 to 267 ng/g. Seven (28%) and two (8%) compounds exhibited acceptable whole and free brain concentrations, respectively. Optimization of maximum free brain levels, IC90s (as a measure or potency) and residual plasma concentrations at 24 h (as a surrogate for half-life) in the same molecule may be feasible since they were not correlated. Diamine quinoline methanols were the most promising lead compounds.

**Conclusion:**

Reduction of CNS levels of NGQMs relative to mefloquine may be feasible. Optimization of this property together with potency and long half-life may be feasible amongst diamine quinoline methanols.

## Background

The Walter Reed Army Institute of Research and collaborators are attempting to identify next generation quinoline methanols for intermittent preventive treatment (IPT) of malaria. IPT is the prevention of morbidity or mortality due to malaria through the intermittent administration of a single dose treatment of a drug at full therapeutic doses to asymptomatic, otherwise healthy infants (IPTi), pregnant women (IPTp) and travelers (IPTt) [1-3]. Drugs for IPTx indications and prophylaxis should ideally exhibit a long half-life, be very well-tolerated and safe in pregnancy. Mefloquine exhibits two of these characteristics, but will likely not find use as an IPT drug because of the adverse CNS events observed at the treatment level doses [4] that may be required for IPT. However, this would presumably not be an issue for next generation analogs of mefloquine without such a liability. Mefloquine accumulates in the CNS and has multiple CNS targets (see discussion in earlier papers [5,6]). The goal is to identify a lead compound for IPT, based on a mefloquine scaffold, for which accumulation into the CNS is substantially reduced. Such a compound should have an improved CNS safety profile relative to mefloquine.

In an earlier study, it was demonstrated that non-piperidine quinoline methanols, in which the piperidine group of mefloquine was replaced with a diamine side chain, were metabolically stable, exhibited reasonable potency against *Plasmodium falciparum in vitro *and were less permeable across MDCK cell monolayers than their monamine counterparts [7,8]. That study did not attempt to address whether reductions in brain concentration relative to mefloquine could be achieved *in vivo*. This was the goal of the present study, in which approximately 25 compounds from our original library were resynthesized, and brain and plasma concentrations were measured over 24 h in mice after i.v. dosing. Plasma concentrations were measured to generate a preliminary indication of half-life, brain concentrations to assess potential exposure relative to mefloquine, and IC90s to assess intrinsic activity against *P. falciparum*.

The dose-limiting CNS side effects of mefloquine at the full therapeutic doses required for IPT include dizziness, incoordination, anxiety and sleeplessness [9]. These common side effects are largely absent at the weekly dose of mefloquine, which is five-fold lower than the treatment dose [10]. Therefore, assuming linearity of mefloquine pharmacokinetics in humans, it makes sense that, assuming no change in affinity for the putative CNS receptors of mefloquine, a five-fold reduction in CNS total drug levels would be the minimum requirement to reasonably expect an improvement in the therapeutic index of a NGQM delivering efficacy at blood exposure equivalent to mefloquine. However, as reported elsewhere [7,8], the lipophilicity of diamine quinoline methanols and other early lead chemotypes is lower than mefloquine. Conceivably this might alter non-specific binding in the brain leading to an increase in the free brain concentration of the drug. Since we do not know the relevant clinical CNS target(s) of mefloquine and the importance of the total and free brain concentration in relation to adverse effects, it is important that reduction relative to mefloquine be assessed with respect to both parameters.

## Methods

### Library synthesis and physicochemical properties

Racemic mefloquine and its (+) and (-) erythro enantiomers were obtained from the WRAIR chemical inventory system. A sub-library of 25 next generation quinoline methanols was synthesized as described in our earlier papers [7,8]. Compounds were almost all rule of 5 (RO5) compliant [11] and designed to encompass the widest feasible range of physiochemical properties (see Table 1). All physiochemical properties were calculated using ACD (Version 10, ACD Labs, Toronto, Canada) except for LogD (pH 7.4) which was determined using Pipeline Pilot. (Version 6.1, Accelrys, San Diego, California). These properties have been defined in detail elsewhere [12], and numerically represent the size (MW), lipophilicity (LogD and LogP), molecular flexibility (FRBs) and H-bonding capacity (HBDs and HBAs) of a compound, all of which impact biological properties important in drug development. The importance of these properties in drug discovery generally and to next generation quinoline methanols more specifically is discussed elsewhere [8]. Given that the focus of this study was on brain penetration, the sub-library of compounds was selected such that a wide range of brain levels was expected based on their physiochemical properties.

**Table 1:** Ranges of physiochemical properties of quinoline methanols investigated in this study.

Parameter	Range
MW	324-475
cLogD (PP)	1.0-5.2
cLogD (ACD)	-2.3-3.1
PSA (Å^2^)	36-104
LogP	0.4-3.7
# FRBs	3-11
# HBDs	1-5
# HBAs	3-7

### Animals, dosing and collection of samples

All *in vivo *studies were conducted in accordance with protocols approved by the Victorian College of Pharmacy, Animal Ethics Committee. Male, six week old FVB mice were obtained from the Animals Resources Center (Perth, Western Australia) and provided with food and water *ad libitum*. Mice were administered a 50 μL bolus injection of mefloquine, its enantiomers or one of twenty five quinoline methanol compounds by tail vein injection (dosing solution concentration 2.8 mg/ml) for a nominal dose of 5 mg/kg. The compounds were prepared in 5% DMSO in 5% glucose solution buffered to a pH of 3-5 using citrate or acetate as appropriate based on an assessment of the lipophilicity and pKa of the compound. At 5 min, 1 h, 6 h or 24 h post-dosing, mice were anaesthetized with inhaled isoflurane (3%) approximately 3-4 minutes prior to blood and brain harvest. Blood was collected by cardiac puncture from groups of 2-3 mice and the whole brain was removed. The whole brains were placed into preweighed polypropylene vials and snap frozen prior to storage along with the plasma samples at -20°C until analysis. On the day of analysis, the whole brain was homogenized in three parts of aqueous solution containing 0.1 M EDTA and 0.4 g/L KF (inhibitors of hydrolytic enzymes to reduce the potential for *ex vivo *tissue-mediated degradation of the test compounds). Calibration standards were prepared by spiking blank brain homogenate and blank plasma with the test compound. Both samples and standards were processed by adding acetonitrile (to precipitate proteins) and centrifuged to separate the supernatant for analysis. An aliquot of the supernatant from each brain homogenate and plasma samples were analysed by UPLC/MS/MS on either a Quattro Premier XE, a Quattro Ultima Pt or a Xevo XT Mass Spectrometer (Waters Corporation, USA).

### Determination of unbound brain fractions by equilibrium dialysis and free brain levels

The unbound fraction of each analog in mouse brain homogenate was determined *in vitro *using equilibrium dialysis according to methodology reported previously [13]. In brief, a 96 well equilibrium dialysis apparatus was used to determine the unbound fraction in brain homogenate for each compound (HT Dialysis LLC, Gales Ferry, CT). Membranes (12-14 kDA cut-off) were conditioned in deionized water for 60 minutes, followed by conditioning in 80:20 deionized water:ethanol for 20 minutes, and then rinsed in artificial cerebrospinal fluid (CSF) before use. Mouse brain was removed from the freezer and allowed to thaw on the day of experiment. Thawed brain tissue was then homogenized with artificial CSF to a final composition of 1:2 brain:artificial CSF using a Covaris S2 (K Biosciences, Hoddesdon, UK). Diluted brain homogenate was then spiked with the test compound (10 ug/g), and 150 μL aliquots (n = 6 replicate determinations) loaded into the 96-well equilibrium dialysis plate. Dialysis vs artificial CSF (150 μL) was carried out for 5 hours in a temperature controlled incubator at *ca*. 37°C (Barworld Scientific Ltd, UK) using an orbital microplate shaker at 125 revolutions/minute (Barworld scientific Ltd, UK). At the end of the incubation period, aliquots of brain homogenate or artificial CSF were transferred to micronic tubes (Micronic B.V., the Netherlands) and the composition in each tube balanced with control fluid, such that the volume of artificial CSF to brain was the same. Sample extraction was performed by the addition of 400 μL of acetonitrile containing an appropriate internal standard. Samples were allowed to mix for 1 minute and then centrifuged at 3000 rpm in 96-well blocks for 15 minutes (Allegra X12-R, Beckman Coulter, USA). All samples were then analysed by UPLC/MS/MS on a Quattro Premier XE Mass Spectrometer (Waters Corporation, USA). The unbound fraction in brain was determined as the ratio of the peak area in artificial CSF to that in brain, with correction for dilution factor according to eq.1 [14].(1)

where D = dilution factor in brain homogenate and f_u,apparent _is the measured free fraction of diluted brain homogenate.

Free brain levels were determined from measured whole brain concentrations and the undiluted f_u _as follows:(2)

### Determination of apparent permeability across MDCK cell layers

Permeability was determined by Absorption Systems (Exton, PA). MDR1-MDCK cell monolayers were grown to confluence on collagen-coated, microporous, polycarbonate membranes in 12-well Costar Transwell plates. Data were considered valid for a specific assay plate if TEER values were < 1400 Ω.cm^2^, the P_app _of propanolol was between 10-30 × 10^-6 ^cm/s and the P_app _of atenolol was < 0.5 × 10^-6 ^cm/s. The permeability assay buffer was Hanks Balanced Salt Solution containing 10 mM HEPES and 15 mM glucose at a pH of 7.4. A known p-glycoprotein inhibitor, cyclosporin A (CSA), was also added to the assay buffer at 10 μM. This was omitted for amphoteric compounds. Bovine serum albumin (1%) was added to the receiver well. The dosing solution concentrations of the test compounds were 5.0 μM in the assay buffer. All cell monolayers were first pre-incubated for 30 minutes with assay buffer. After 30 minutes, the buffer was removed, replaced with fresh buffer, and time was recorded as 0. The addition of BSA, pre-incubation, and use of a longer incubation time were employed to mitigate potential low recovery or permeability that is sometimes observed for lipophilic or 'sticky' compounds. Cell monolayers were dosed on the apical side (A-to-B) or basolateral side (B-to-A) and incubated at 37°C with 5% CO2 in a humidified incubator. After two hours, aliquots were taken from the receiver chambers. Samples were taken from the donor chamber at 0 and 2 hours. Each determination was performed in duplicate. The lucifer yellow flux was also measured for each monolayer to assess monolayer integrity during the flux period. All samples were assayed by LC/MS/MS using electrospray ionization.

Apparent permeability in the apical (A-B direction), Papp_A-B_, and percent recovery are reported in the supplementary Information. Apparent permeability is a measure of the rate of transport across the cell monolayer. Percent recovery refers to the amount of compound recoverable at the end of the assay. Low recovery may indicate non-specific binding to assay plates, instability or accumulation in the cell pellet. In the case of mefloquine, relatively low recovery is likely a consequence of accumulation in cell membranes [15,16] rather than non-specific binding.

The apparent permeability, Papp, and percent recovery were calculated as follows:(3)(4)

where,

*d*Cr/*d*t is the slope of the cumulative concentration in the receiver compartment versus time in μM s^-1^.

Vr is the volume of the receiver compartment in cm^3^.

Vd is the volume of the donor compartment in cm^3^.

A is the area of the cell monolayer (1.13 cm^2 ^for 12-well Transwell).

C0 is the measured concentration of the donor chamber at time 0 in μM.

CN is the nominal concentration of the dosing solution in μM.

Cr^final ^is the cumulative receiver concentration in μM at the end of the incubation period.

Cd^final ^is the concentration of the donor in μM at the end of the incubation period.

### *Plasmodium falciparum *susceptibility assays

The *in vitro *activities of quinoline methanols against *P. falciparum *strains W2, D6, TM91C235, and TM90C2A were evaluated using the traditional labeled hypoxanthine assay of Desjardins *et al *[17] as modified by Milhous *et al *[18]. These four *P. falciparum *strains were selected since they have various levels of resistance to conventional anti-malarials. W2 is chloroquine resistant and mefloquine sensitive, D6 is chloroquine sensitive but naturally less susceptible to mefloquine, TM91C235 is resistant to mefloquine, chloroquine, and pyrimethamine as is TM90C2A, however this latter parasite is a two *pfmdr1 *copy strain (pfmdr1 amplification has been associated with clinical mefloquine resistance). Mefloquine is routinely screened in these assays to ensure the validity. Historical values for mefloquine against all the strains are reported elsewhere [8]. The focus of this study was the mefloquine-sensitive strain W2, however values for the other strains for all the compounds are reported in the Supplementary Information.

### Data analysis

Prism graphing software was used to graph key datasets in order to facilitate visual inspection for possible correlations amongst parameters of interest followed by statistical confirmation if needed. For graphical analysis of IC90 data, IC90s were assumed to be equivalent to the maximum concentrations tested where they were insufficiently potent to be determined. Our brain uptake studies yielded plasma concentration data for four time points. This was insufficient for the purposes of determining half-lives in most instances. As a surrogate for half-life, we determined the % residual plasma concentrations at 24 h versus concentrations at 5 min. Where the measured plasma concentration was below the analytical lower limit of quantitation (LLQ), an arbitrary value of 1 ng/ml was assigned for the purposes of analysis. For analysis of MDCK permeability data versus maximum brain concentration, we did not adjust Papp values on the basis of % recovery. However, percent recovery values are included in the Supplementary Information. The probability of a diamine exhibiting the desired free brain concentration, IC90s and residual plasma concentration was calculated by multiplying the marginal probabilities of each of these events since they appeared to be independent. The +/- 95% confidence limits for the probability estimate was calculated using the formula: +/- 95% CIs = 1.96*SQRT(P*(1-P)/n) where P is probability of success and SQRT is the square root of the result of the function in brackets.

## Results

**Figure 1:**
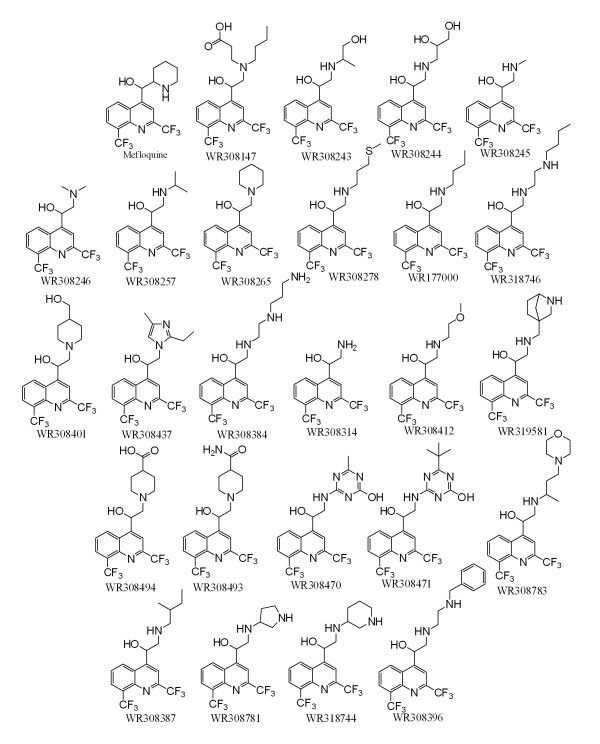
**Structures of quinoline methanols investigated in this study**. The structure of racemic mefloquine is indicated. Its two diasteromers were also investigated in this study.

Twenty-five quinoline methanols were successfully resynthesized or obtained from the WRAIR chemical inventory system. Their structures are depicted in Figure 1. Physicochemical properties, *in vitro *susceptibility data, brain tissue binding data, brain and plasma concentrations and MDCK screening data are summarized in Additional file 1. The range of physicochemical properties encompassed by the mini-library of compounds is summarized in Table 1.

**Figure 2:**
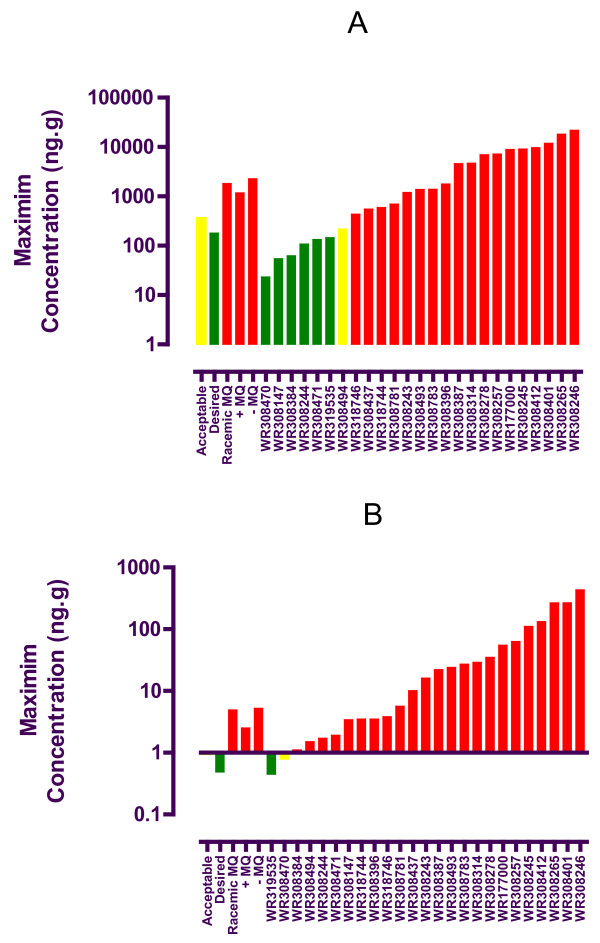
**Maximum whole (A) and free (B) brain concentrations for racemic mefloquine, + *erythro *mefloquine, (-) *erythro *mefloquine and 25novel quinoline methanols after IV dosing to male FVB mice (5 mg/kg)**. Compounds are color-coded green, yellow or red based on whether they exhibited the desired (> ten-fold lower than racemic mefloquine), acceptable (> five-fold lower than racemic mefloquine) or unacceptable brain levels, respectively.

The maximum whole and free brain concentrations of the quinoline methanols are summarized in Figures 2A and 2B, respectively. The maximum whole and free brain concentrations of racemic mefloquine were 1807 and 4.9 ng/g. For the (+) and (-) mefloquine enantiomers, these values were (whole/free) 1159/2.5 and 2249/5.2 ng/g, respectively. The lowest whole brain concentration was 23 ng/g (WR318470) and the lowest free brain concentration was 0.45 ng/g (WR319535). Of the 25 novel compounds, 7 (28%) and 2 (8%) passed the minimum acceptable five-fold reduction in whole and free brain levels, respectively.

**Figure 3:**
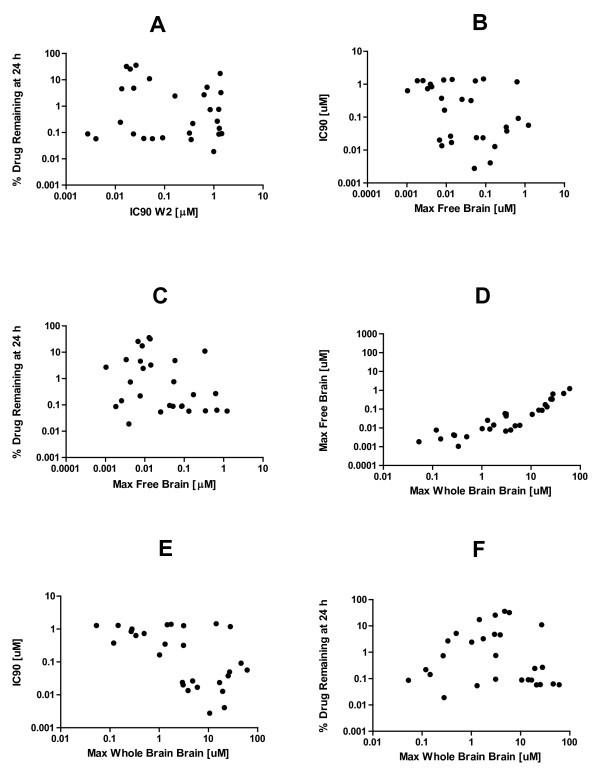
**Relationships amongst four important pharmacological parameters for quinoline methanols were evaluated for evidence of correlations**. A possible linear correlation between maximum and free brain concentration is evident on visual inspection of the data, and was confirmed mathematically (r^2 ^= 0.89, P < 0.0001). There was no relationship evident between the other variables. Data are presented in a log format for ease of visualization. The correlation coefficient was calculated based on raw (not log transformed) data.

Pharmacological data for four important parameters were examined for evidence of possible correlations in order to justify assumptions regarding probability estimates and to assess the feasibility of the optimization of desirable traits in a single molecule. There was no evidence of correlations amongst the following parameters: residual plasma concentrations at 24 h, maximum free brain concentration and IC90s (Figures 3A-C). This suggests that (i) their probability distributions may be independent and (ii) they may be optimizable in the same molecule. There was an apparent linear relationship evident between maximum whole and free brain concentrations (Figure 3D), that was confirmed mathematically (r^2 ^= 0.89, *P *< 0.0001, linear regression). This suggests that these variables are not independent and that 89% of the variability in free brain concentrations can be explained by variability in whole brain concentrations alone. There was no qualitative evidence of a simple linear correlation between IC90 and maximum whole brain concentration (Figure 3E). However, the lack of data in the lower left quadrant suggests more caution in making the assumption of independence between these two variables. There was no evidence of correlation between residual drug concentrations and maximum whole brain concentration (Figure 3F).

**Figure 4:**
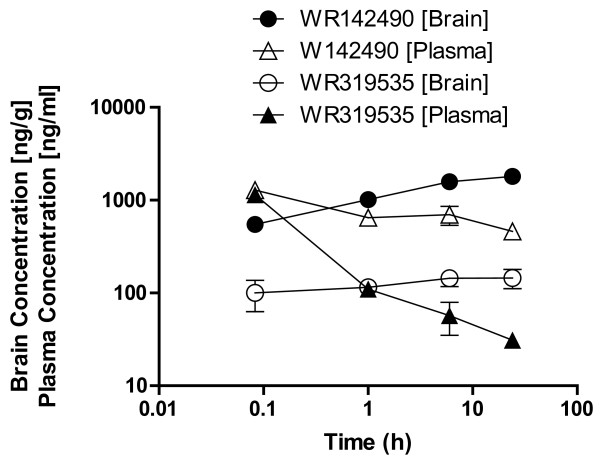
**Brain and plasma concentrations of racemic mefloquine and the diamine WR319581 after IV dosing (5 mg/kg) to male FVB mice**.

**Figure 5:**
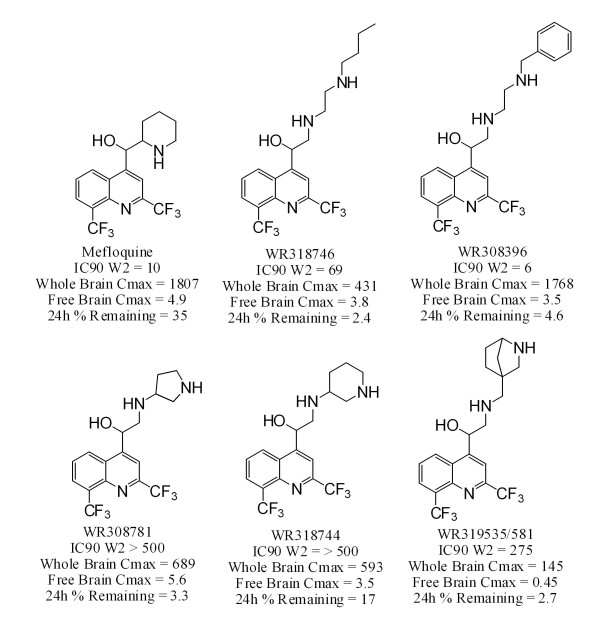
**Structures, IC90s, maximum whole and free brain concentrations, and residual 24 h plasma concentrations of diamine quinoline methanols**. IC90s are expressed in ng/ml against the mefloquine-sensitive W2 strain of *P. falciparum*. Maximum whole and free brain concentrations in male FVB mice after iv dosing (5 mg/kg) are expressed in ng/g. Residual plasma concentrations at 24 h after iv dosing are expressed as a percentage relative to the 5 minute time point. One in 5 diamines (20%, WR308396) had acceptable potency. One in 5 diamines had acceptable whole (20%, WR319535) and free (20%, WR319535) brain levels relative to mefloquine. WR319535 exhibited a half-life longer than mefloquine after oral dosing (data not shown). For this reason we have assumed that the residual plasma concentrations at 24 h of all (100%) of the diamines may be indicative of long half-lives.

One of the most promising compounds was WR319535 (Figures 1, 2 and 4). This compound exhibited maximum whole and free brain concentrations of 145 and 0.45 ng/g, respectively. Significant drug levels remained at 24 h post-dosing (Figure 4) compared to many of the other compounds investigated (Supplementary Information) which may imply a long half-life. Unfortunately, the compound exhibited only modest potency. Four related diamine quinoline methanols (Figure 5) were also evaluated. When these were considered together, 1 in 5 exhibited appropriate potency, 1 in 5 exhibited appropriate whole brain levels, and 1 in 5 exhibited appropriate free brain levels relative to mefloquine. All of these compounds were represented in the group of 9 non-piperidine compounds with residual plasma concentrations > 1% of the observed maximum value. This suggests they represent the sub-chemotype with the greatest propensity for a long half-life. The probability (+/- 95% confidence intervals) of such a molecule exhibiting the desired free brain levels, potency and residual plasma concentrations was estimated to be 4 (0-21)% (Figure 5).

The apparent permeability (A-B) of quinoline methanols was used to categorize them as having either higher (if Papp was > 7.4 × 10^-6 ^cm/s) or lower (if Papp was < 7.4 × 10^-6 ^cm/s) maximum brain concentrations than mefloquine *in vivo *(Table 2). Compounds with apparent permeability values of < 7.4 × 10^-6 ^cm/s exhibited lower brain concentrations than mefloquine *in vivo *in 100% of cases. Compounds with apparent permeability > 7.4 × 10^-6 ^cm/s exhibited maximum brain concentrations higher than mefloquine in 71% of cases (Table 2). Thus, is it is likely that an MDCK permeability acceptability threshold of < 7.4 × 10^-6 ^cm/s would exclude most future compounds for which maximum brain concentrations are higher than mefloquine.

**Table 2:** Utility of MDCK permeability screen for ranking maximum brain concentrations of quinoline methanols relative to mefloquine.*

	Compounds with Papp < 7.4 × 10^-6 ^cm/s	Compounds with Papp > 7.4 × 10^-6 ^cm/s
Compounds with lower maximum brain concentrations than mefloquine	10	4
Compounds with higher maximum brain concentrations than mefloquine	0	10

* Racemic mefloquine and isomers excluded

## Discussion

The focus of the WRAIR discovery program with next generation quinoline methanols has been to reduce partitioning into the CNS since the relevant clinical target or targets of mefloquine adverse events are not known [19]. The incidence of the most common adverse CNS effects of mefloquine is reduced substantially when the dose is lowered five-fold from the treatment to the weekly prophylaxis level. Presumably this is also associated with a five-fold decline in CNS levels as well. Consequently, at least a five-fold reduction in whole and free brain levels relative to mefloquine at the efficacious dose is probably the minimum requirement. In this study we have shown that at least a ten-fold reduction in both parameters is feasible through replacement of the piperidine ring of mefloquine with various other four-position substituents. The prospect of such a compound being better tolerated than mefloquine is, therefore, a possibility.

While a reduction in maximum brain concentration should reduce the incidence of CNS associated adverse events, optimizing this property alone will not be sufficient for a new quinoline methanol anti-malarial. The putative late lead compound must also have a sufficiently long half-life and intrinsic efficacy to be useful if administered as a single dose. Therefore, the goal is to balance these three potentially competing pharmacological properties in the same molecule. There is an opportunity to evaluate a subset of the larger 4-position library using surrogates of these endpoints. Maximum free and whole brain concentrations represent surrogates of CNS safety if they are sufficiently reduced relative to mefloquine at the therapeutic dose. Whilst the plasma concentration-time data generated are insufficient to calculate pharmacokinetic parameters directly in most instances, the proportion of drug remaining at 24 h relative to the 5 minute time point is probably a reasonable surrogate of half-life, or at least persistence in plasma. *In vitro *activity (IC90) can be utilized as a surrogate for intrinsic activity.

There are no compounds amongst this sub-library that meet all the requirements in terms of potency, low brain levels and residual plasma concentrations at 24 h (see Additional file 1) suggesting that further optimization is required. The ideal chemotype around which to anchor a future lead optimization program would be one in which a balance between all these three properties is achievable. The most active compounds were generally those with aliphatic, ether or thio ether side chains (WR308245, WR308246, WR308257, WR308265, WR177000, WR308387, WR308278 and WR308412). However, these compounds exhibited much higher brain concentrations (free and whole) than mefloquine, and in most instances, 24 h residual plasma concentrations were < 1% of those at 5 min, indicating a likely short half-life relative to mefloquine. The exception to this general trend was the diamine WR308396. As might be expected, all compounds with lower whole and free brain concentrations than mefloquine had a greater number of H-bond donors and/or acceptors than mefloquine. These compounds comprised a diverse array of structures including alcohols, acids, triazines, substituted cyclic imidazoles and diamines. However, with the exceptions of the diamines WR319581 and WR318746, these compounds exhibited a lack of potency (IC90 > 300 ng/ml) or low residual plasma concentrations (< 1% at 24 h versus 5 min). Of the 25 compounds that were not mefloquine or its enantiomers, only nine had residual plasma concentrations > 1% at 24 h, and five of these were diamines. Consequently, a balance between these properties may be most likely obtained amongst diamine quinoline methanols.

Identifying a new lead compound with the requisite balance of potency, brain partitioning and half-life might not be feasible if it was true that positive trends in these characteristics were mutually exclusive, however this does not appear to be the case. Maximum free brain concentrations, IC90s, and residual plasma concentrations amongst quinoline methanols did not appear to be correlated (Figure 3A-C). Therefore, at the outset there is no reason to suspect that a balance between these properties is not achievable in a diamine quinoline methanol. There are no generic algorithms that can be used to objectively judge the probability of success of a specific lead optimization programme *a priori*. Thus one must extrapolate on a project-by-project basis with the available data. The lack of a general correlation between maximum free brain concentrations, residual plasma concentrations and IC90s suggests that their probability distributions may be independent. Thus, we estimated the probability of a diamine quinoline methanol possessing all these properties to be around 4%. This value was obtained by multiplying the marginal probabilities of success for the individual parameters of interest (Figure 5). The confidence intervals are wide (0-21%) which is not surprising given the small sample sizes.

The probability estimate discussed above was based on free brain concentrations being representative of CNS exposure. We have not made the same calculations for maximum whole brain concentrations, because the assumption of independence between this variable and IC90 may arguably be less justifiable. However, two lines of evidence support the notion that a compound with an acceptable maximum free brain concentrations will also have an acceptable maximum whole brain concentrations. First, whole and free brain concentrations in the broader group of quinoline methanols were strongly correlated. This suggests that they are also not independent, or put another way, that there is a strong conditional probability whole brain concentrations will be lower than mefloquine if this is the case for free brain concentrations. Second, in the same broader group of quinoline methanols it is also true that the proportion of compounds exhibiting the desired whole brain concentrations (28%) was higher than the proportion of compounds exhibiting the desired free brain concentrations (8%). Additional studies are required to determine whether these observations will hold for a larger sample of diamine quinoline methanols.

It is appropriate to execute a lead optimization programme if it can be done with a reasonable probability of success in a time frame of up to two years [20]. For planning purposes, it has been assumed the true probability of success for an individual compound lies somewhere between the lower confidence interval and 4%. Since the synthetic route is amenable to the synthesis of 100 new compounds in 12-18 months, it is anticipated that up to four potential late lead molecules will be identified in that time frame. The assumed low probability of success with any individual compound requires an aggressive method of early triage. The permeability of a compound across MDCK cell monolayers appears to correctly categorize it relative to mefloquine in terms of its maximum brain concentration in most instances. It is, therefore, reasonable to use this assay together with more routine *P. falciparum *susceptibility assays to rapidly prioritize compounds for in *vivo *studies. It may be necessary to conduct *in vivo *studies on a selection of active, but permeable compounds triaged using this technique in order to control the type II error rate. A lead optimization campaign conducted broadly along these lines is now underway.

## Competing interests

The authors declare that they have no competing interests.

## Authors' contributions

All authors made contributions to this study through past or current membership of the Next Generation Quinoline Methanol project team. The objective of this project team is to identify a development candidate from the quinoline methanol class for malaria prophylaxis and IPT. GSD, KR and SC conceived the general project study. GSD analysed the data and prepared the manuscript. EEM, WFM, SG and JM synthesized the analogs. NR and LG determined IC90s. DC determined LC50s. SN performed the equilibrium dialysis. JS, JSC, DS, KW, QZ and SC coordinated and/or executed the brain uptake experiments. JB determined the physiochemical properties. All authors read and approved the final manuscript.

## Supplementary Material

Additional File 1 **Structures, physiochemical properties and biological data for a collection of diamine quinoline methanols**. This file contains the structures of 25 diamine quinoline methanols, racemic mefloquine and its isomers, their key physiochemical properties, and biological data from Pf in vitro screening, cytotoxicity screening, MDCK permeability studies, plasma and brain binding experiments, and brain and plasma concentration data after i.v. dosing in mice. The methods are described in the text of the manuscript. Click here for file
